# Enablement and empowerment among patients participating in a supported osteoarthritis self-management programme – a prospective observational study

**DOI:** 10.1186/s12891-022-05457-9

**Published:** 2022-06-08

**Authors:** Karin Sturesdotter Åkesson, Anne Sundén, Kjerstin Stigmar, Cecilia Fagerström, Teresa Pawlikowska, Eva Ekvall Hansson

**Affiliations:** 1 Department of Health Sciences, Lund University, Lund, Sweden; 2 Department of Research and Education, Skåne University Hospital, Lund, Sweden; 3 Department of Health and Caring Sciences, Linnæus University, Kalmar, Sweden; 4 Health Professions Education Centre, RCSI University of Medicine and Health Sciences, Dublin, Ireland

**Keywords:** Physiotherapist, Patient education, Osteoarthritis, Patient partner, Enablement, Empowerment, Primary health care

## Abstract

**Background:**

In Sweden, core treatment for osteoarthritis is offered through a Supported Osteoarthritis Self-Management Programme (SOASP), combining education and exercise to provide patients with coping strategies in self-managing the disease. The aim was to study enablement and empowerment among patients with osteoarthritis in the hip and/or knee participating in a SOASP. An additional aim was to study the relation between the Swedish version of the Patient Enablement Instrument (PEI) and the Swedish Rheumatic Disease Empowerment Scale (SWE-RES-23).

**Methods:**

Patients with osteoarthritis participating in a SOASP in primary health care were recruited consecutively from 2016 to 2018. The PEI (score range 0–12) was used to measure enablement and the SWE-RES-23 (score range 1–5) to measure empowerment. The instruments were answered before (SWE-RES-23) and after the SOASP (PEI, SWE-RES-23). A patient partner was incorporated in the study. Descriptive statistics, the Wilcoxon’s signed rank test, effect size (r), and the Spearman’s rho (r_s_) were used in the analysis.

**Results:**

In total, 143 patients were included in the study, 111 (78%) were women (mean age 66, SD 9.3 years). At baseline the reported median value for the SWE-RES-23 (*n* = 142) was 3.6 (IQR 3.3–4.0). After the educational part of the SOASP, the reported median value was 6 (IQR 3–6.5) for the PEI (*n* = 109) and 3.8 (IQR 3.6–4.1) for the SWE-RES-23 (*n* = 108). At three months follow-up (*n* = 116), the reported median value was 6 (IQR 4–7) for the PEI and 3.9 (IQR 3.6–4.2) for the SWE-RES-23. The SWE-RES-23 score increased between baseline and three months (*p* ≤ 0.000). The analysis showed a positive correlation between PEI and SWE-RES-23 after the educational part of the SOASP (*r*_*s*_ = 0.493, *p* < 0.00, *n* = 108) and at follow-up at three months (*r*_*s*_ = 0.507, *p* < 0.00, *n* = 116).

**Conclusions:**

Patients reported moderate to high enablement and empowerment and an increase in empowerment after participating in a SOASP, which might indicate that the SOASP is useful to enable and empower patients at least in the short term. Since our results showed that the PEI and the SWE-RES-23 are only partly related both instruments can be of use in evaluating interventions such as the SOASP.

**Trial registration:**

ClinicalTrials.gov. NCT02974036. First registration 28/11/2016, retrospectively registered.

**Supplementary Information:**

The online version contains supplementary material available at 10.1186/s12891-022-05457-9.

## Background

Worldwide, osteoarthritis (OA) is a common joint disease that causes pain, disability and decreased health-related quality of life [1]. Prevalence is expected to increase since the number of aging and obese people is growing [2, 3] and also due to the fact that there is no contemporary cure for OA [4, 5]. The global burden of this disease is huge [3] and the costs for health care are increasing [2, 6]. First-line evidence-based treatment for OA is patient education, exercise and, if needed, weight loss [7–9].

The World Health Organization (WHO) recommends patient education as part of the management of all patients with chronic disease, including OA [10]. Patient education is defined as “helping patients acquire or maintain the competencies they need to manage as well as possible their lives with a chronic disease. It is an integral and continuing part of patient care. It comprises organised activities, including psychosocial support, designed to make patients aware of and informed about their disease and about health care, hospital organization and procedures, and behavior related to health and disease, so that they (and their families) understand their disease and their treatment, collaborate with each other and take responsibility for their own care as a means of maintaining or improving their quality of life” ([10], p. 17). In Sweden, patients with OA in the hip and/or knee are offered first-line treatment through a Supported Osteoarthritis Self-Management Programme (SOASP) [11, 12]. Self-management can be defined as “the individual’s ability to manage the symptoms, physical treatment, psychological consequences, and lifestyle changes inherent in living with a chronic condition” ([13], p. 547) and the SOASP aims to provide the patients with coping strategies and knowledge to support in self-managing the disease [11, 14]. Swedish OA patients participating in a SOASP are offered to report data in a national quality register called “Better Management of patients with Osteoarthritis” (BOA) [14]. The BOA register evaluates for example pain, health-related quality of life and physical activity that supports improvement of treatment for patients with OA in the hip and/or knee [11]. Today, to our knowledge, patients´ ability to cope and self-manage their disease is not evaluated routinely neither after participating in a SOASP nor reported in the BOA register.

The WHO has recognised that health care should make more effort to enable and empower patients with chronic disease [10, 15, 16]. Enablement and empowerment are closely related concepts [17–19]. As evaluated by the Patient Enablement Instrument (PEI), patient enablement is defined as patients´ ability to understand and cope with their illness after a consultation in health care [20, 21]. The WHO defines empowerment as “a process through which people gain greater control over decisions and actions affecting their health” and “Individual empowerment refers primarily to the individuals’ ability to make decisions and have control over their personal life” ([22], p. 6). Empowerment can be measured by the Swedish Rheumatic Disease Empowerment Scale (SWE-RES-23) [23].

Taking all these aspects into account it seems important to increase knowledge about how patients experience their ability to self-manage their OA. To our knowledge, there are no studies about enablement or empowerment in relation to patients with OA after participating in a SOASP. This study can contribute with more knowledge on relevant evaluation methods. Therefore, the aim was to study enablement and empowerment among patients with OA in the hip and/or knee participating in a SOASP. An additional aim was to study the relation between the Swedish version of the PEI and the SWE-RES-23.

## Methods

### Design and setting

We conducted a prospective observational study, using data from patients with OA in the hip and/or knee participating in a SOASP in primary health care (PHC). The study was approved by the Regional Ethical Review Board in Lund, Sweden (2015/918). The study was reported in accordance with the STROBE checklist [24] and was registered with ClinicalTrials.gov. NCT02974036, first registration 28/11/2016, retrospectively registered.

### The supported osteoarthritis self-management programme

According to existing national guidelines, patients diagnosed with OA are to be offered participation in a SOASP in relation to getting diagnosed. The programme combines education and exercise and is often provided by a physiotherapist (PT) in PHC [12]. The SOASP usually consists of two to three educational sessions once a week providing the patients with information about OA, risk factors, symptoms, treatment, coping strategies and self-management [12]. After the educational part of the programme, patients are offered an individually adapted exercise programme that they can practice either at home or in a group training, supervised by a PT for about 6 to 8 weeks [12]. The SOASP has been described in more detail elsewhere [12].

### Participants, data collection and measurements

**Fig. 1:**
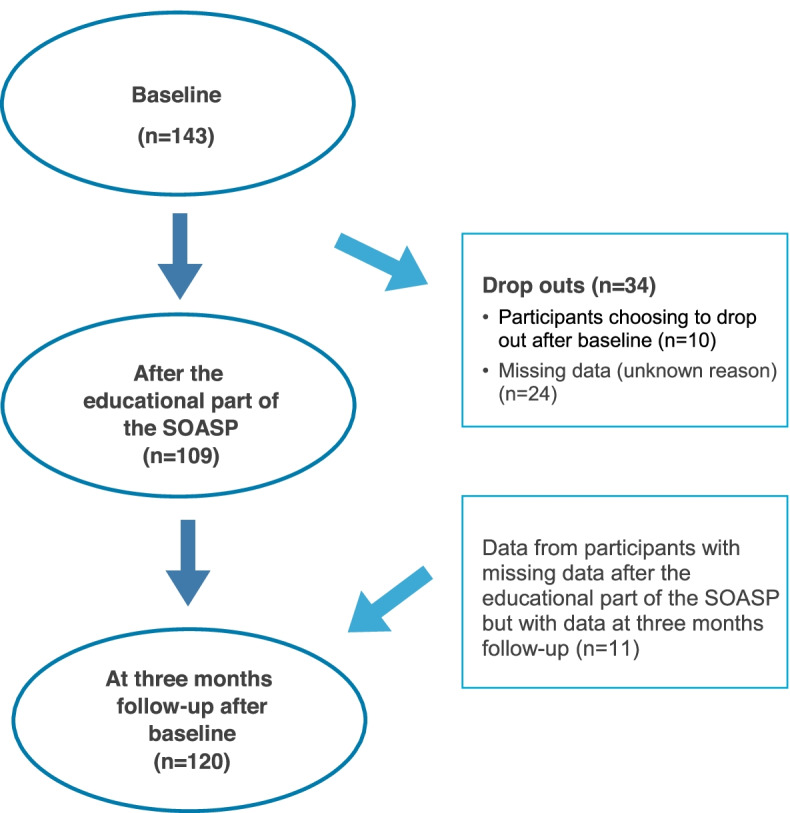
Flowchart for the collection of participants for analysis in the study

Data was collected in PHC in two health care regions in southern Sweden: Region Skåne (five PHC centres, *n* = 87) and in Region Blekinge (two PHC centres, *n* = 56) between April 2016 and June 2018. Inclusion criteria in the study were patients with hip and/or knee OA understanding Swedish and participating in the SOASP. There were no exclusion criteria. Patients participating in a SOASP were recruited consecutively and asked to participate in the study by the PT responsible for the SOASP at the PHC centre in question. All patients that were interested in participating in the study were given written and verbal information about the study and gave their written informed consent for study participation prior to the start of the study. Flowchart for the inclusion of participants for analysis in the study is presented in Fig. 1.

Patient reported outcome measures (PROMs) were answered at baseline (SWE-RES-23), after the educational part of the SOASP and at three months follow-up (PEI and SWE-RES-23). The Patient Enablement Instrument (PEI) (Additional file 1) was used to measure enablement [20, 21, 25]. The PEI was developed in the 1970´s with the aim to measure a patient´s ability to understand and cope with their disease after a consultation [20, 21, 25]. The PEI consists of six questions relating to an introductory sentence “As a result of your visit to the doctor today, do you feel you are…”. In our study, the introductory sentence was changed to “As a result of your participation in the SOASP, do you feel you are…”. Each question had four alternative answers, i.e., much better (scored 2), better (scored 1), same or less (scored 0), not applicable (scored 0), resulting in a total consultation score between 0 to 12 [20, 21, 25]. A higher total score indicates higher enablement [20, 21, 25]. There is no baseline data reported for the PEI as the instrument is based on the patients´ own perception of change in enablement after a consultation [20].

Empowerment was measured with the Swedish Rheumatic Disease Empowerment Scale (SWE-RES-23) [23] (Additional file 2) that has been developed from the Swedish Diabetes Empowerment Scale [23]. The SWE-RES-23 consist of 23 questions divided in five subscales. The questions 1 to 3 start with “In terms of how I take care of my rheumatic disease, I…”, the questions 4 to 7 start with “In terms of my rheumatic disease, I…”, questions 8 to 11 start with, “In terms of my rheumatic disease, I…” and, questions 12 to 23 start with, “In general, I think I…”. In our study, the word “rheumatic disease” was replaced with the word “osteoarthritis”. Each question is scored on a five-point Likert scale ranging from strongly disagree (1 point) to strongly agree (5 points). The total score is calculated by summating the score of each question and dividing the sum by 23, resulting in a total score between 1 to 5 points where a higher score indicates higher empowerment [23].

Both the PEI and the SWE-RES-23 have been translated to Swedish and have been tested for reliability [23, 26] and for validity [23, 27]. The PEI has shown high internal consistency and moderate to good reliability [26] whereas content validity, construct validity and internal consistency was fair [27]. The SWE-RES-23 has shown acceptable psychometric properties, in terms of construct validity and internal consistency reliability [23].

### Patient partner

To enhance the patient perspective, a patient partner (PP) from the Swedish Rheumatism Association was involved in the study process from the beginning. The PP contributed with feedback on the aim of the study, feasibility of the study approach, the PROMs used in the study and assisted in interpreting the results. We first met with the PP face to face at a network event lasting three days to plan the study, discuss the aim and the feasibility of the study approach and to practically test the PROMs, estimate the time to answer them and discuss their relevance in the context of the SOASP. We met once more physically when the study was ongoing to discuss preliminary results. Thereafter, we met digitally thrice, and we kept contact through email. The GRIPP-2 checklist was used when reporting the PP´s involvement in the study process (Additional file 3) [28, 29].

### Statistical analysis

Descriptive statistics (median and interquartile range (IQR)) were used to describe the degree of enablement and empowerment patients with OA in the hip and/or knee report after the educational part of the SOASP and at three months follow-up after participating in a SOASP.

The Wilcoxon’s signed rank test was used when analysing the significance of the change in the SWE-RES-23 from baseline to three months follow-up. The effect size for the change in the SWE-RES-23 from baseline to three months follow-up, based on the Wilcoxon’s signed rank test, was computed according to the formula *r* = Z / √ N [30, 31] and categorized as small (0.1), medium (0.3) or large (0.5) [32].

The Spearman’s rho (*r*_*s*_) was used when analysing the relation between the PEI and the SWE-RES-23 after the educational part of the SOASP and at three months follow-up. The correlation values were categorised as weak (0.1–0.3), moderate (0.3–0.5) or strong (0.5 or more) [32]. The median, IQR and non-parametric tests were used in the analysis since the PEI and SWE-RES-23 scales were treated as ordinal scales. A sample size calculation showed that to be able to detect a correlation coefficient between 0.3 to 0.5 with a power of 0.80 at a chosen significance level of 0.05, 110 participants were needed. The calculation was performed in SAS Enterprise Guide 6.1 for Windows (SAS Institute Inc., Cary, NC, USA). Data from 143 participants were collected to compensate for potential missing data. No imputation was made for missing values [33].

## Results

In total, 143 patients agreed to participate and were included in the study, 111 (78%) were women (mean age 66, SD 9.3 years). Demographic data for the study cohort (*n* = 143) are presented in Table 1.

**Table 1:** Sample characteristics

	**Study sample (*n* = 143)**
Gender % (*n*)	
Men	22 (32)
Women	78 (111)
Age (years)	
mean (SD)	65.9 (9.3)
min–max	40–90
Most affected joint % (*n*)	
knee	72.1 (101)
hip	25.7 (36)
hand	2.1 (3)
missing data	2.7 (3)
BMI^*^	
mean (SD)	28.9 (6.3)

^*^BMI = Body Mass Index (*n* = 135)

Information from 143 patients at baseline, from 109 patients after the educational part of the SOASP and from 120 patients at follow-up at three months was collected (Fig. 1). Ten patients (7%) dropped out after baseline due to unknown reasons.

After the educational part of the SOASP, the reported median value was 6 (IQR 3–6.5) for the PEI (*n* = 109) and 3.8 (IQR 3.6–4.1) for the SWE-RES-23 (*n* = 108). At three months follow-up (*n* = 116), the reported median value was 6 (IQR 4–7) for the PEI and 3.9 (IQR 3.6–4.2) for the SWE-RES-23.

The Wilcoxon’s signed rank test revealed a statistically significant increase in empowerment from baseline to three months follow-up, *Z* = -4.07, *p* ≤ 0.000 (*n* = 115), with an effect size close to medium (*r* = 0.27).

**Fig. 2:**
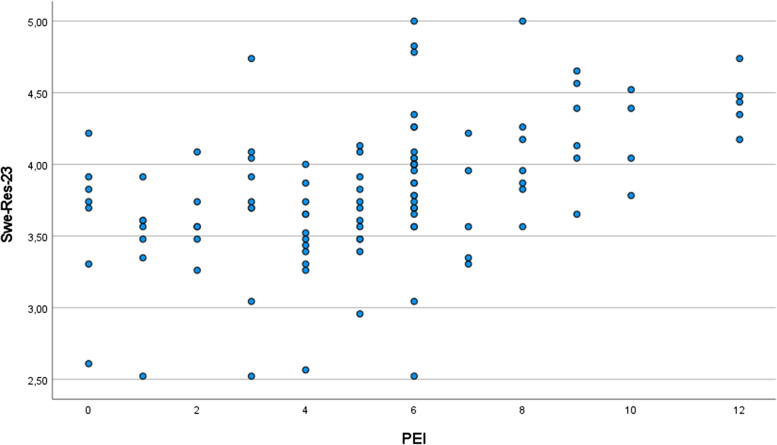
Scatterplot showing the correlation between the PEI and the SWE-RES-23 after the educational part of the SOASP (Spearman’s rho (*r*_*s*_) = 0.493; *n* = 108)

**Fig. 3:**
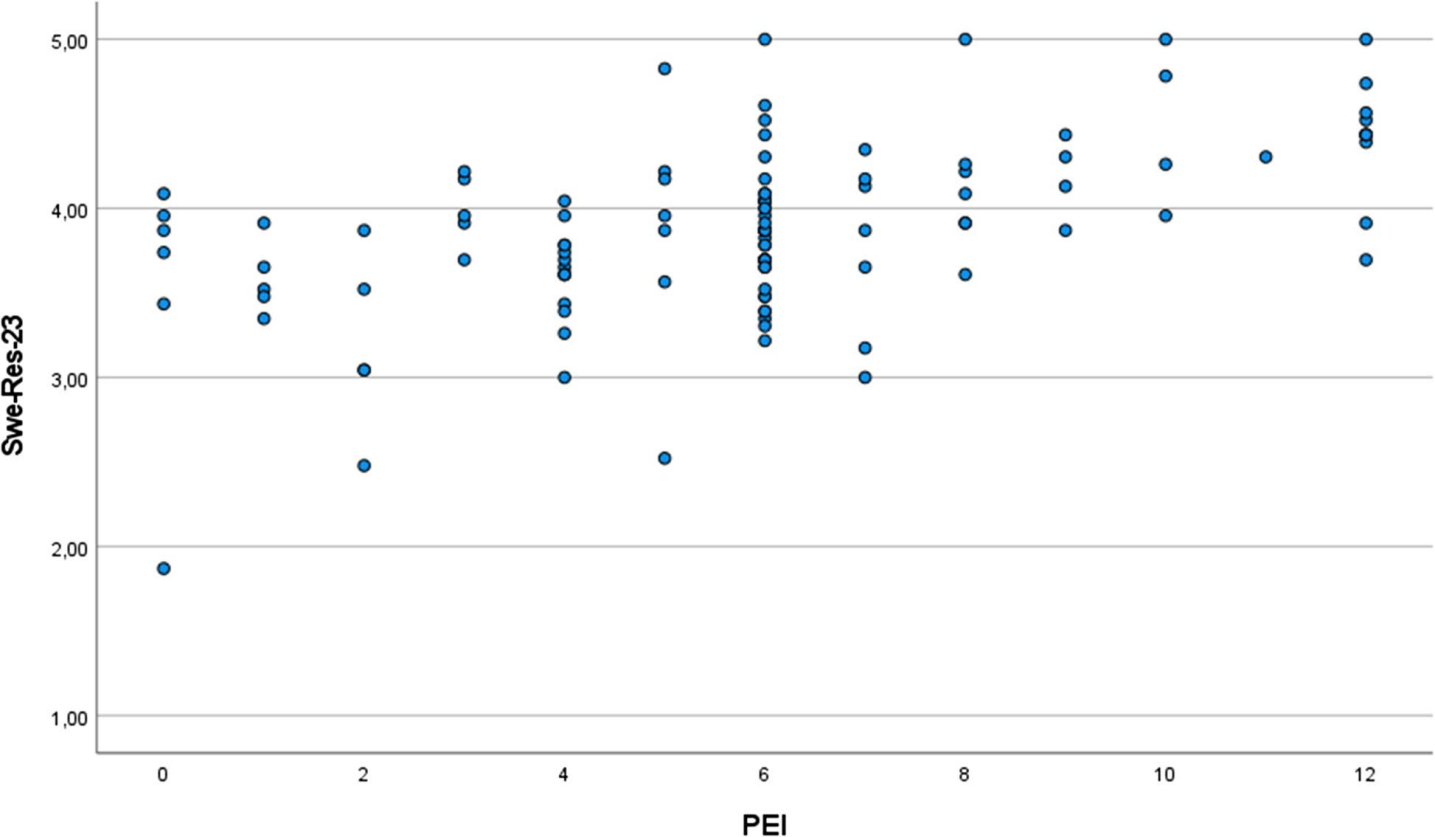
Scatterplot showing the correlation between the PEI and the SWE-RES-23 at follow-up at three months (Spearman’s rho (*r*_*s*_) = 0.507; *n* = 116)

The analysis showed positive correlation between PEI and SWE-RES-23 both after the educational part of the SOASP (r_s_ = 0.493, *p* < 0.00, *n* = 108) (Fig. 2) and at follow-up at three months (r_s_ = 0.507, *p* < 0.00, *n* = 116) (Fig. 3). Both correlations were close to cut-off point (0.5) for strong correlation.

## Discussion

Our study shows that patients with OA report having moderate to high enablement and empowerment both after the educational part of the SOASP and at three months follow-up after participating in a SOASP. Moreover, there was a statistically significant increase in empowerment at three months after participation in the SOASP. In addition, the relation between the PEI and the SWE-RES-23 was close to the cut-off point for strong correlation both after the educational part of the SOASP and at three months follow-up.

To our knowledge, neither the PEI nor the SWE-RES-23 have been used in a similar setting i.e., patient education for OA before [25]. However, in a Swedish validation study of the PEI, three groups of patients with musculoskeletal disorders were included [27]. One group included patients with chronic pain that were referred to a multimodal rehabilitation programme [27]. The programme lasted 6 to 8 weeks and included pain education and exercise [27]. The authors analysed the median value of the PEI after the programme which was reported to be 3 [27]. This is considerably lower than in our study where the median PEI value at three months follow-up in our study was 6. This lower PEI score has been reported for patients with severe pain [34] and for patients with three or more chronic diseases [35].

The patients reported moderate to high values on both the PEI and the SWE-RES-23 at both measuring points. It may be that participants in a SOASP might be more informed and motivated per se since they seek health care for their problems and accept to participate in an active intervention such as the SOASP. Thus, there might be a selection bias when it comes to which patients with OA participate in the SOASP in comparison to the total population with hip and/or knee OA in Sweden, which is an issue that has been raised in previous studies on patients with OA in relation to SOASP [36, 37]. This perspective was supported by the PP incorporated in the study who also reflected that patients who seek health care might be more willing to change and to do something about their problems.

In our study, the PEI score was maintained at the same level at the follow-up which is not in line with the results in a study by Rööst et al. [26] where the PEI decreased at follow-ups at two days and two weeks after consultation in PHC. However, the divergence in the results between our study and the study by Rööst et al. might be due to differences in intervention, length of the intervention, what health profession the patient consulted with, and the time point for follow-up. These thoughts were highlighted by the PP and are supported by other researchers [27, 38]. In addition, there is a risk of recall bias since the PEI is based on the patients´ perception of change in enablement which the patients might not recall when answering the questions [27, 39]. Thus, it might be argued that is not known how enabled the patient is [27].

It is not surprising that the scores from both the PEI and the SWE-RES-23 seem to be moderate to high even at three months follow-up since patients that have participated in supervised exercise probably have been continuously encouraged with information and reminded of coping strategies and the importance of exercising. However, comparison with other studies is challenging since there is no global consensus on what a high value in context is either on the PEI [40] or the SWE-RES-23. For the PEI, a total score of 6 or more has been suggested to be high [21]. In a recently published study where the SWE-RES-23 was used, a score of ≥ 4.05 was considered to be high [41]. In relation to these studies, we therefore believe that the results of our study indicate relatively high scores. Moreover, patient education itself is a patient-centered learning process and self-management is about what patients themselves decide to do to manage their treatment and prevent complications [10]. It takes time for an individual to adapt to a new health-condition [10]. Therefore, three months follow-up is a short time when it comes to a chronic disease like OA, and it would be interesting to follow the development of enablement and empowerment after participating in a SOASP in the long-term. In the future, it would also be interesting to study those who report lower values on the PEI and/or the SWE-RES-23 more closely since it might be important to identify these patients as early as possible to optimise the support and care.

Our study showed that empowerment significantly increased after participating in the SOASP which is encouraging. However, if the increase is due to participating in the SOASP needs to be further studied as well as whether the increase is sustainable. To our knowledge, there are no other studies evaluating empowerment in relation to SOASP, thus the results from our study can be used as comparison in future studies.

The PP pointed out that it might be difficult for newly diagnosed patients to answer the PEI and above all question number 4 i.e., “able to keep yourself healthy” directly after the educational sessions.

According to national guidelines, all patients should be offered to participate in SOASP at diagnosis, but that is not always the case in practice. Therefore, some patients might have had their diagnosis for some time, sometimes many years, before participating in SOASP. Reasons for that might vary (patients not wanting to see at PT before, patients not being referred to at PT but to a doctor, all health care professionals not following the guidelines and so on) [42]. This delay in participation in SOASP would affect patients´ adaptation, understanding and coping with their illness and that would impact on their PEI score and their enablement. So, patients participating in a SOASP who have had their diagnosis for a long time might already have some knowledge about coping and might answer the PEI and the SWE-RES-23 differently than a newly diagnosed patient. This is in accordance with other studies [26, 27] that have raised the idea that patients might answer the PEI differently depending on how long they have had their disease. In the future, it would be interesting to study the time relationship between diagnosis and self-reported enablement and empowerment.

Our results showed that the relation between the PEI and the SWE-RES-23 was close to the cut-off point for strong correlation at both measuring points thus, the instruments only partly measure the same entity. Therefore, we believe that this relationship needs to be further investigated. However, the results suggest that both the PEI and the SWE-RES-23 could be useful when evaluating the SOASP, which was supported by the PP incorporated in the study. One might argue that the instrument that the patients find most relevant and valuable should be the one used for evaluation. However, the PP thought that the large amount of data collected in this study show that it is feasible to use both the instruments i.e., patients seem to think that it is acceptable to answer them both.

There seems to be some confusion about the concepts of enablement and empowerment in the literature and the concepts are sometimes used interchangeably [17–19, 39, 43]. This makes comparison with different studies challenging. Enablement occurs after an intervention or consultation in health care [21, 25, 44, 45] while empowerment can be achieved both after an education but also by oneself [46]. Today, enablement and empowerment are not routinely evaluated in relation to SOASP and the PEI and/or the SWE-RES-23 could possibly be used both in the clinic and included in the BOA register in the future to ensure evaluation of these relevant outcomes. However, more research is needed before it can be concluded which of the two outcomes is the most relevant to measure in this context.

## Strengths and weaknesses

A strength with the study was that data was collected by PTs´ used to collecting PROMs in connection to the SOASP which might explain why the response rate was high and the small amount of missing data. Another strength was that a PP was included in the study process from the planning phase of the study to the interpretation of the results. In the planning phase, the PP gave feedback on the PEI and the SWE-RES-23 regarding feasibility to answering them after participating in a SOASP and estimating the time to answer them. The PP also provided valuable input regarding the interpretation of the results and clinical implications. The results and implications were validated by the PP who also added new perspectives based on experiential knowledge of living with OA. Moreover, the PP gave valuable suggestions for future research. Engaging a PP in research was not common in Sweden when planning this study (2015) and there were not many PPs with adequate education available at the time. In future studies, we hope to incorporate more than one PP since we believe that it would enhance the research process considerably.

There are some limitations in our study. The results of our study are difficult to compare with other studies for several reasons. We analysed the median value since both the PEI and the SWE-RES-23 can be considered as Likert scales and thus ordinal data. Other studies have analysed the PEI and the SWE-RES-23 using the mean values [23, 25, 40, 47] and also there are not many studies using the SWE-RES-23 [23]. In addition, the PEI score outcomes vary in different countries [25, 48–50], which make comparison between different studies challenging. In our study, we used the SWE-RES-23 to measure empowerment. Developed in 2012 and thus being a relatively new instrument, it has not been much used or studied. Therefore, an argument might be that we should have used another instrument when measuring empowerment. However, the SWE-RES-23 was developed for rheumatic disease and was in the developing phase tested by patients with OA, which we thought was valuable when planning the study. In this study, we compared a generic instrument i.e., the PEI to a disease specific instrument i.e., the SWE-RES-23 (rheumatic diseases). Generic instruments are developed for measurements in a broad range of populations with or without chronic illness while disease specific instruments are designed for measuring concerns relevant to a particular disease [51]. Unfortunately, as this was an in vivo clinic based study, we have no information about the reasons for dropouts. However, we believe that since distributing the questionnaires after the educational part of the SOASP was added to the clinical routine, some PTs might have forgotten to do so. Moreover, no control group was included, and casual relationships cannot be assessed in our observational study. These limitations are something to keep in mind when interpreting the results.

## Implications

Even though the main objective with the SOASP is to support patients´ ability to cope and self-manage their disease this is not routinely evaluated after participating in a SOASP today. We find it important to evaluate patient enablement and empowerment after participating in a SOASP and therefore we suggest using the PEI and/or the SWE-RES-23 together with the PROMs that are used currently.

We believe that including a PP in the study process from the planning phase to the interpretation of the results enhances the constructive learning experience of health care professionals and researchers drawn from the study and we highly recommend other researchers to incorporate a PP in their studies.

## Conclusions

Patients reported moderate to high enablement and empowerment and an increase in empowerment after participating in a SOASP, which might indicate that the SOASP is useful to enable and empower patients with OA in the hip and/or knee at least in the short term. Since our results showed that the PEI and the SWE-RES-23 are only partly related, we believe that both instruments can be of use in evaluating interventions such as the SOASP depending on the outcome of interest.

## Supplementary Information

**Additional file 1.** The Patient Enablement Instrument (PEI).

**Additional file 2.** The Swedish Rheumatic DiseaseEmpowerment Scale (SWE-RES-23).

**Additional file 3.** GRIPP2 short form.

**Additional file 4.** Summary plain language long version.

**Additional file 5.** Summary plain language short version.

## Data Availability

The dataset generated and analysed during the current study are not publicly available due to the ethics approval and Swedish law (but are available from the corresponding author on reasonable request).

## Abbreviations

OA: Osteoarthritis
WHO: World Health Organization
SOASP: Supported Osteoarthritis Self-Management Programme
BOA: Better Management of patients with Osteoarthritis
PEI: Patient Enablement Instrument
SWE-RES-23: Swedish Rheumatic Disease Empowerment Scale
PHC: Primary Health Care
PT: Physiotherapist
PROM: Patient Reported Outcome Measure
PP: Patient Partner
IQR: Interquartile Range
