# Supplementary figures and images for: Enablement and empowerment among patients participating in a supported osteoarthritis self-management programme – a prospective observational study
Source: BMC Musculoskelet Disord. 2022 Jun 8;23:555. doi: 10.1186/s12891-022-05457-9 (PMC9175380; doi:10.1186/s12891-022-05457-9)

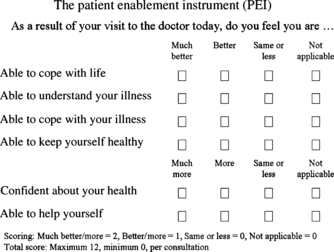

Supplement: Supplementary file 1 — Additional file 1. The Patient Enablement Instrument (PEI). [file 12891_2022_5457_MOESM1_ESM.docx]
