# Supplementary material for: Enablement and empowerment among patients participating in a supported osteoarthritis self-management programme – a prospective observational study
Source: BMC Musculoskelet Disord. 2022 Jun 8;23:555. doi: 10.1186/s12891-022-05457-9 (PMC9175380; doi:10.1186/s12891-022-05457-9)
Supplement: Supplementary file 2 — Additional file 2. The Swedish Rheumatic DiseaseEmpowerment Scale (SWE-RES-23). [file 12891_2022_5457_MOESM2_ESM.doc]

| **In terms of how I take care of my rheumatic disease, I…** | Strongly agree | Agree | Neutral | Disagree | Strongly disagree |
| --- | --- | --- | --- | --- | --- |
| 1… know what parts of self-care that causes dissatisfaction | 5 | 4 | 3 | 2 | 1 |
| 2… know what parts of self-care that I am ready to change | 5 | 4 | 3 | 2 | 1 |
| 3… know what parts of self-care that I am not ready to change | 5 | 4 | 3 | 2 | 1 |

| **In terms of my rheumatic disease…** | Strongly agree | Agree | Neutral | Disagree | Strongly disagree |
| --- | --- | --- | --- | --- | --- |
| 4… I think I mostly can choose realistic rheumatic disease goals | 5 | 4 | 3 | 2 | 1 |
| 5… I think I mostly know what helps in reaching rheumatic disease goals | 5 | 4 | 3 | 2 | 1 |
| 6… I think I mostly know how to reach my rheumatic disease goals | 5 | 4 | 3 | 2 | 1 |
| 7…I think I mostly can turn goals into a workable plan | 5 | 4 | 3 | 2 | 1 |

| **In terms of my rheumatic disease, I think I usually....** | Strongly agree | Agree | Neutral | Disagree | Strongly disagree |
| --- | --- | --- | --- | --- | --- |
| 8… know what barriers that make reaching rheumatic disease goals more difficult | 5 | 4 | 3 | 2 | 1 |
| 9… can overcome barriers to reaching goals | 5 | 4 | 3 | 2 | 1 |
| 10… can try out different ways of overcoming barriers | 5 | 4 | 3 | 2 | 1 |
| 11… can decide the best way of overcoming barriers | 5 | 4 | 3 | 2 | 1 |

| **In general, I think I…** | Strongly agree | Agree | Neutral | Disagree | Strongly disagree |
| --- | --- | --- | --- | --- | --- |
| 12… know how having a rheumatic disease causes stress in life | 5 | 4 | 3 | 2 | 1 |
| 13… know positive ways of coping with rheumatic disease-related stress | 5 | 4 | 3 | 2 | 1 |
| 14… know negative ways of coping with rheumatic disease-related stress | 5 | 4 | 3 | 2 | 1 |
| 15… cope well with rheumatic disease-related stress | 5 | 4 | 3 | 2 | 1 |
| 16… know where to get support for caring for my rheumatic disease | 5 | 4 | 3 | 2 | 1 |
| 17… ask for support for caring for rheumatic disease when needed | 5 | 4 | 3 | 2 | 1 |

| **In general, I think I…** | Strongly agree | Agree | Neutral | Disagree | Strongly disagree |
| --- | --- | --- | --- | --- | --- |
| 18… can support myself in dealing with rheumatic disease | 5 | 4 | 3 | 2 | 1 |
| 19… know how to stay motivated to take care for the rheumatic disease | 5 | 4 | 3 | 2 | 1 |
| 20… know how to motivate myself to take care for the rheumatic disease | 5 | 4 | 3 | 2 | 1 |
| 21… know enough to make self-care choices | 5 | 4 | 3 | 2 | 1 |
| 22… know enough about myself as a person to make rheumatic disease care choices | 5 | 4 | 3 | 2 | 1 |
| 23… can think about if it is worth my while to change how to take care of the rheumatic disease | 5 | 4 | 3 | 2 | 1 |

Reference: Arvidsson, S., Bergman, S., Arvidsson, B., Fridlund, B., & Tingström, P. (2012). Psychometric properties of the Swedish rheumatic disease empowerment scale, SWE-RES-23. *Musculoskeletal Care, 10*(2),101-9. doi: 10.1002/msc.1005.

Each question in each factor is summarized individually and the sum is divided by the number of items.

To calculate the overall mean SWE-RES-23 score, the value of all items are summarized and the sum is divided by 23.

A higher value indicates a stronger empowerment.

- Factor 1,”Goal achievement and overcoming barriers to goal achievement”, includes the questions 4, 5, 6, 7, 8, 9, 10 and 11.
- Factor 2, “Self-knowledge”, includes the questions 18, 19, 20, 21, 22 and 23.
- Factor 3, “Managing stress”, includes the questions 12, 13, 14 and 15.
- Factor 4, “Assessing dissatisfaction and readiness to change”, includes the questions 1, 2 and 3.
- Factor 5, “Support for caring”, includes the questions 16 and 17.
