# Supplementary material for: Enablement and empowerment among patients participating in a supported osteoarthritis self-management programme – a prospective observational study
Source: BMC Musculoskelet Disord. 2022 Jun 8;23:555. doi: 10.1186/s12891-022-05457-9 (PMC9175380; doi:10.1186/s12891-022-05457-9)
Supplement: Supplementary file 3 — Additional file 3. GRIPP2 short form. [file 12891_2022_5457_MOESM3_ESM.docx]

**GRIPP2 short form**

| **Section and topic** | **Item** | **Reported on page No** |
| --- | --- | --- |
| 1: Aim | Report the aim of PPI in the study | 4 |
| 2: Methods | Provide a clear description of the methods used for PPI in the study | 4 |
| 3: Study results | Outcomes—Report the results of PPI in the study, including both positive and negative outcomes | 5–7 |
| 4: Discussion and conclusions | Outcomes—Comment on the extent to which PPI influenced the study overall. Describe positive and negative effects | 5–7 |
| 5: Reflections/critical perspective | Comment critically on the study, reflecting on the things that went well and those that did not, so others can learn from this experience | 7–8 |
