# Supplementary material for: Enablement and empowerment among patients participating in a supported osteoarthritis self-management programme – a prospective observational study
Source: BMC Musculoskelet Disord. 2022 Jun 8;23:555. doi: 10.1186/s12891-022-05457-9 (PMC9175380; doi:10.1186/s12891-022-05457-9)
Supplement: Supplementary file 4 — Additional file 4. Summary plain language long version. [file 12891_2022_5457_MOESM4_ESM.docx]

**Patients benefit from participating in a** **Supported Osteoarthritis Self-Management Programme**

*In a Supported Osteoarthritis Self-Management Programme you learn about osteoarthritis,* *what you can do to cope with the disease and you get support in how to be physically active. Education and exercise are the core treatment for osteoarthritis, but we need to know that the programme is well received and useful. This is what our research is about.*

Osteoarthritis is a common disease globally and estimates show that even more people will be affected in the future. Any joint can be affected by osteoarthritis, but it is most commonly found in the spine, hip, knee, and fingers. If you have osteoarthritis, you can have pain when being physically active, you might feel stiff and be unable to perform activities in everyday life like standing up, walking stairs, vacuum-cleaning or to button your shirt depending on what joint is affected.

Today, there is no cure for osteoarthritis but there is still a lot you can do to ease the burden of the disease to feel as good as possible. Since osteoarthritis is a common disease, there is a lot of research on the it which has shown that all people with osteoarthritis should all have information about the disease and get support in how to be physically active, moreover, to lose weight if necessary.

All patients with osteoarthritis should be offered participation in a supported osteoarthritis self-management programme, preferably as early as possible in the process of the disease. When participating in the programme you will learn about osteoarthritis and how to be physically active. It is important that you know what you yourself can do to be able to cope with the disease. If you start being physically active early in the disease process you could maintain your mobility, increase muscle strength, and ease pain. Regular proper exercise might prevent impairment.

Our study shows that people with osteoarthritis in the hip and/or knee report being better able to cope with their illness after participation in a supported osteoarthritis self-management programme. They also report that their belief in their own capacity to manage everyday life increase after the programme. This is promising since it is aligned with the aim of the programme. Two questionnaires that patients with osteoarthritis answered themselves were used to evaluate the programme. We worked with a patient partner who has experience of living with osteoarthritis and so added new perspectives to our study.

It is important that the supported osteoarthritis self-management programme is of high quality and that those living with osteoarthritis learn how to cope with the disease. Then those affected are enabled to ease pain, maintain physical function, and avoid impairment.

Enablement and empowerment among patients participating in a Supported Osteoarthritis Self-Management Programme – A prospective observational study

Karin Sturesdotter Åkesson^a^*, Anne Sundén^a^, Kjerstin Stigmar^ab^, Cecilia Fagerström^c^, Teresa Pawlikowska^d^, Eva Ekvall Hansson^a^.

*^a^Department of Health sciences, Lund University, Lund, Sweden; ^b^Department of Research*

*and Education, Skåne University Hospital, Lund, Sweden; ^c^Department of Health and Caring*

*Sciences, Linnæus University, Kalmar, Sweden; ^d^Health Professions Education Centre, RCSI*

*University of Medicine and Health Sciences, Dublin, Ireland.*

*corresponding author: Karin.sturesdotter_akesson@med.lu.se
